# Functional roles of Arabidopsis *CKRC2/YUCCA8* gene and the involvement of PIF4 in the regulation of auxin biosynthesis by cytokinin

**DOI:** 10.1038/srep36866

**Published:** 2016-11-09

**Authors:** Dong-Wei Di, Lei Wu, Li Zhang, Chen-Wei An, Tian-Zi Zhang, Pan Luo, Huan-Huan Gao, Verena Kriechbaumer, Guang-Qin Guo

**Affiliations:** 1 Institute of Cell Biology and MOE Key Laboratory of Cell Activities and Stress Adaptations, Lanzhou University, Lanzhou 730000, China; 2 Plant Cell Biology, Biological and Medical Sciences, Oxford Brookes University, Oxford OX3 0BP, United Kingdom

## Abstract

Auxin and cytokinin (CK) are both important hormones involved in many aspects of plant growth and development. However, the details of auxin biosynthesis and the interaction between auxin and CK are still unclear. Isolation and characterization of an auxin deficient mutant *cytokinin induced root curling 2 (ckrc2*) in this work reveal that *CKRC2* encodes a previously identified member of YUCCA (YUC) flavin monooxygenase-like proteins (YUC8). Our results show that, like other YUCs, CKRC2/YUC8 is a rate-limiting enzyme for catalyzing the conversion of indole-3-pyruvic acid (IPyA) to indole-3-acetic acid (IAA), acting downstream of CKRC1/TAA1 in the IPyA pathway. Here we show that the transcription of both *CKRC1/TAA* and *CKRC2*/*YUC8* can be induced by CK and that the phytochrome-interacting factor 4 (PIF4) is required for this upregulation. Transcription of *PIF4* itself is induced by CK via the AHKs-ARR1/12 signalling pathway. These results indicate that PIF4 plays an essential role in mediating the regulatory effect of CK on the transcriptions of *CKRC1* and *CKRC2* genes in the IPyA pathway of auxin biosynthesis.

Auxin is an important phytohormone and influences many processes in plant growth and development, such as cell division and elongation, tropism, apical dominance, senescence and blooming^1^,^2^,^3^,^4^,^5^. *In planta*, auxin homeostasis is controlled by biosynthesis, transport and metabolism^6^. Based on biochemical and genetic evidence, it has been proposed that IAA, the predominant form of auxin in plants, is synthesized via two major pathways: Trp-dependent (TD) and Trp-independent (TI) pathways^5^,^7^,^8^. The TD pathway can further be divided into four branch pathways according to their first intermediate metabolites, i.e., the indole-3-acetamide (IAM) pathway, the indole-3-pyruvic acid (IPyA) pathway, the tryptamine (TAM) pathway and the indole-3-acetaldoxime (IAOx) pathway. So far only the IPyA pathway has been completely determined on genetic and biochemical levels. For many years, no gene or intermediate metabolite involved in the TI pathway has been identified. Most recently, however, careful studies on the mutant of the indole synthase (INS) gene provide evidence that the cytoplasmic protein INS is involved in auxin biosynthesis via the TI pathway^9^.

*YUC* genes were initially linked to auxin biosynthesis based on the finding that overexpression of *YUC1* leads to an auxin overproduction phenotype^10^. There are 11 predicted members of *YUC* genes encoding YUCCA (YUC) flavin monooxygenase-like proteins in *Arabidopsis*. Overexpression of each of the *YUCs* results in high auxin phenotypes^11^. However, inactivation of a single *YUC* gene does not cause obvious developmental defects suggesting overlapping functions among *YUC* genes^11^,^12^. YUC1 was initially suggested to catalyze the conversion of TAM to N-hydroxylated tryptamine (HTAM) in the TAM pathway^10^ but recent studies have placed the YUC proteins downstream of CKRC1/TAA1, catalyzing the conversion of IPyA to IAA^13^,^14^,^15^,^16^. Further results showed that YUC can synthesize a quasi-stable 4-α-hydroperoxyl flavin intermediate from flavin adenine dinucleotide (FADH-) and acts on numerous substrates^12^,^17^,^18^^. It was reported that YUC6 utilizes NADPH and O_2_ to convert IPyA to IAA^^12^. In this work we show that, like other YUCs, CKRC2/YUC8 is a rate-limiting enzyme in the IPyA pathway for catalyzing the conversion of IPyA to IAA. Together with CKRC1/TAA1, CKRC2/YUC8 plays an essential role in the CK-dependent regulation of auxin biosynthesis.

The interaction between auxin and CK plays a key role in plant growth and development^19^. Recent studies reveal that CK can regulate both the biosynthesis and the polar transport of auxin via its signaling pathway^19^,^20^,^21^. We previously reported that CK can stimulate auxin biosynthesis by up-regulating the transcription of *CKRC1/TAA1* and other auxin biosynthesis genes including *YUC8*^21^; however, the associated transcriptional factors/regulators have not been identified so far. Here we show that PIF4 is essential for CK-dependent regulation of *CKRC1/TAA1* and *CKRC2/YUC8* transcription.

## Results and Discussions

### Comparison of root phenotypes among mutants in different *YUC* genes and their transcription

**Figure 1:**
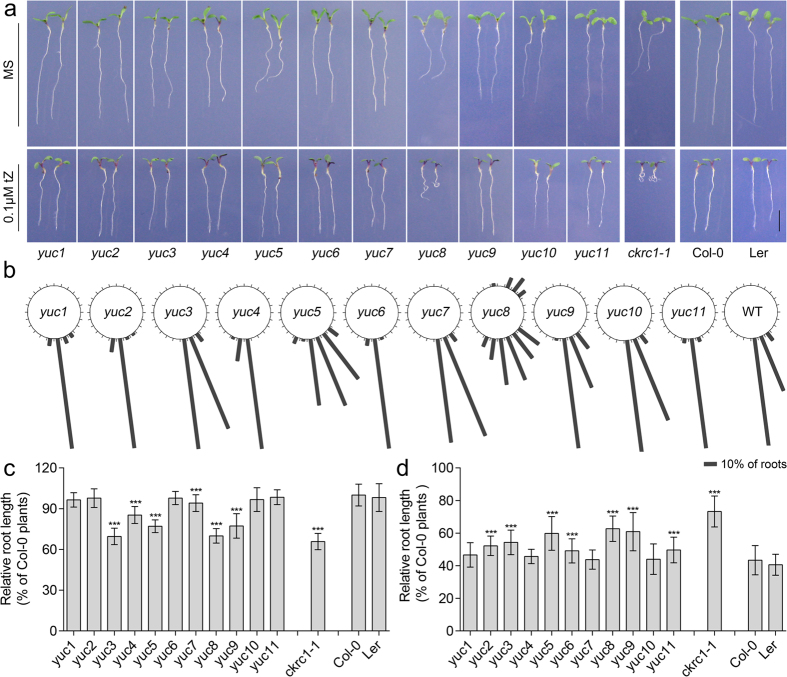
Comparison of root phenotypes between *yuc mutants*. (**a**) The phenotypes of all 11 *yuc* mutants on MS medium with (bottom) or without (top) 0.1 μM tZ 7d after germination (bar = 5 mm); (**b**) Gravitropic responses of *yuc* mutants. Seedlings were grown on MS medium for 6d, then transferred to fresh MS medium and reoriented 90 degrees for 24 hours (n = 75–100); (**c**,**d**) Root elongation was measured 7d after germination on MS with (**c**) or without (**d**) 0.1 μM tZ. All experiments were repeated 3 times. Shown are mean values ± SD with n = 40–45 in each repeat. ***P < 0.001.

The *ckrc2-1* mutant was isolated as one of the auxin-deficient mutants in a large-scale forward genetic screen for the so-called *cytokinin induced root curling* (*ckrc*) mutants (Supplementary Fig. S1)^21^,^22^. When grown on medium containing 0.1 μM trans-zeatin (tZ) these mutants display a root curling phenotype. Genetic and molecular analysis identified *ckrc2-1* as a loss of function mutation in the *YUC8* gene. The mutation is caused by a 3554 bp deletion in the promoter coding region (Supplementary Fig. S2). As *YUC8* is one of 11 members of the *YUC* gene family functioning in auxin biosynthesis^10^,^11^,^12^,^13^,^14^,^15^,^16^, root phenotypes in the other 10 *YUC* genes were also analysed (Fig. 1a & Supplementary Fig. S3). We found that none of the single mutants in other *YUC* genes had the *ckrc2*/*yuc8*-like curling root phenotype on the medium containing 0.1 μM tZ (Fig. 1a). Furthermore only *yuc8* displayed a significantly defective gravitropic response (GR) on MS medium. *Yuc5*, showed a weak root GR defect (Fig. 1a,b). Root measurements showed that most *yuc* mutants had decreased root length when grown on MS medium (Fig. 1c), and *yuc5*, *ckrc2-1/yuc8* and *yuc9* were less sensitive to 0.1 μM tZ in terms of relative root length compared to other *yuc* mutants (Fig. 1d).

**Figure 2:**
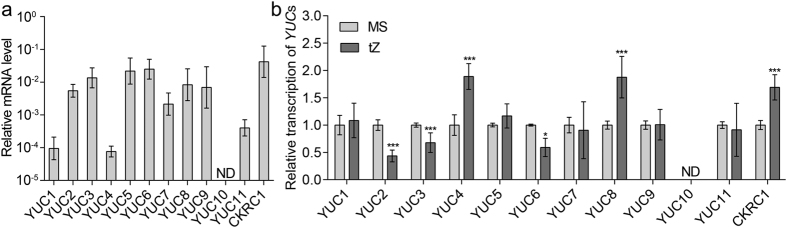
*CKRC2/YUC8* is highly transcribed in roots and induced by tZ. (**a**) Analysis of the relative transcription of *YUC* genes in roots by qRT-PCR; (**b**) Analysis of the relative transcription of *YUC* genes after 0.1 μM tZ treatment. Data are mean values of 3 replicates. Error bars indicate ± SD. 0.05 > *P > 0.01; ***P < 0.001.

To determine why only *ckrc2-1/yuc8* showed a root curling phenotype, the relative transcription of *YUC* genes in roots and whole seedlings was analyzed by qRT-PCR (Fig. 2a & Supplementary Fig. S4). Consistent with data previously reported by Chen *et al.*^23^, *YUC3, YUC5, YUC7, YUC8* and *YUC9* were highly expressed in roots (Fig. 2a & Supplementary Figs S4 and S5). However, in our results also *YUC2* and *YUC6* were detected in high levels in roots (Fig. 2a & Supplementary Fig. S4). Analysing the relative transcription of the *YUC* gene family after tZ treatment, we found that out of the seven *YUC* genes with high transcription levels in roots, only *CKRC2/YUC8* showed significant up-regulation by tZ (Fig. 2b). Up-regulation of the transcription of *CKRC2/YUC8* after short time treatment with tZ was previously also shown in microarray data and qRT-PCR results ( http://www.weigelworld.org/resources/microarray/AtGenExpress/) (Supplementary Figs S6 and S7).

The high abundance of *YUC8* in roots and its up-regulation by CK could explain why *ckrc2-1/yuc8* is the only *yuc* single mutant with a curled roots phenotype when grow on tZ containing medium and thus could be isolated in our CK forward genetic screen.

### *CKRC2/YUC8* encodes an enzyme catalyzing a rate-limiting step in the IPyA pathway for IAA biosynthesis

**Figure 3:**
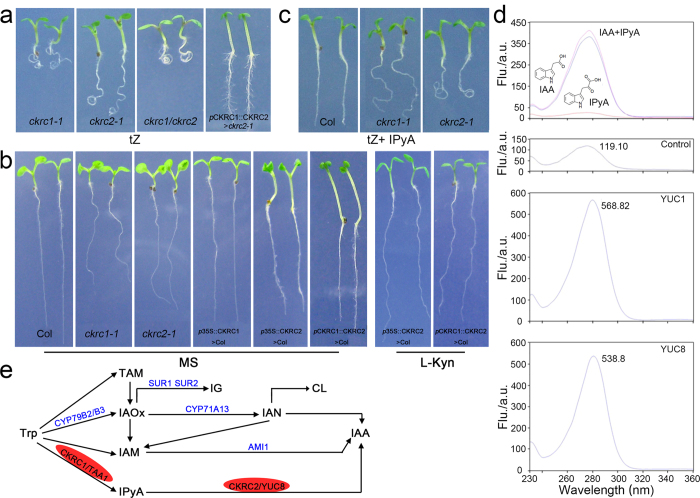
CKRC2/YUC8 and CKRC1/TAA1 function in the same biosynthetic pathway and CKRC2/YUC8 catalyzes a rate-limiting step. (**a**) The *ckrc1-1 ckrc2-1* double mutant displays a less-than-additive phenotype and the curling root phenotype of *ckrc2-1* on tZ medium can be rescued by overexpression of *CKRC2/YUC8* cDNA under the control of the *CKRC1/TAA1* promoter (*p*CKRC1::CKRC2 > *ckrc2-1*); (**b**) The high auxin phenotype of transgenic *p35S::CKRC2* or *pCKRC1::CKRC2* plants can be inhibited by the addition of 1 μM L-Kyn; (**c**) Phenotypic analysis of Col, *ckrc1-1* and *ckrc2-1* plants grown on 0.1 μM tZ medium with 0.01 μM IPyA is shown; (**d**) Both CKRC2/YUC8 and YUC1 catalyze the conversion of IPyA to IAA; (**e**) Schematic diagram of auxin biosynthetic pathways.

Some members of YUC family, including YUC1, YUC2, YUC4 and YUC6, have been shown to function in the same biosynthetic pathway with CKRC1/TAA1 and are catalyzing the conversation of IPyA to IAA, a rate-limiting step in the TAA/YUC pathway^12^,^13^,^14^,^16^,^22^. To determine if this was also the case for YUC8, a double mutant of *ckrc2/yuc8 ckrc1/taa1* was phenotypically compared with the two parent single mutants *ckrc2/yuc8* and *ckrc1/taa1*. In terms of root curling when grown on tZ medium the double mutant showed a less-than-additive phenotype (Fig. 3a & Supplementary Fig. S1), a phenomenon often associated with two mutations in different steps of the same linear genetic pathway^16^,^24^. The curling roots phenotype of *ckrc2-1/yuc8* on tZ medium can be rescued by expressing of *CKRC2/YUC8* cDNA under the control of the *CKRC1/TAA1* promoter (Fig. 3a). Overexpression of *CKRC2*/*YUC8* cDNA under the control of a *35S* or *CKRC1*/*TAA1* promoter Col-0 plants results in high auxin phenotypes such as increased amounts of root hairs, epinastic leaves, and elongated hypocotyls (Fig. 3b)^10^ indicating that CKRC2/YUC8 is catalyzing a rate-limiting step in IAA biosynthesis. This is not the case when overexpressing *CKRC1*/*TAA1* under a 35S promoter in a wildtype background (Fig. 3b)^21^,^25^. Notably, the high-auxin phenotypes in *p35S::CKRC2* or *pCKRC1::CKRC2* transgenic plants can be suppressed by addition of L-kynurenine (L-Kyn), a specific TAA1/TAR inhibitor. This is indicating that the CKRC2/YUC8 mediated IAA production depends on the function of CKRC1/TAA1 (Fig. 3b)^26^. Moreover, addition of endogenous IPyA in tZ medium can partially rescue the curling root phenotype of *ckrc1/taa1* but not that of *ckrc2/yuc8* (Fig. 3c). Those results support the hypothesis that CKRC2/YUC8 and CKRC1/TAA1 act in the same biosynthetic pathway and CKRC2/YUC8 catalyses a rate-limiting step downstream from CKRC1/TAA1.

Recent work has established that in IAA biosynthesis CKRC1/TAA1 catalyzes the conversion of Trp to IPyA and YUCs catalyze the subsequent oxidation of IPyA to IAA^12^,^13^,^14^,^15^,^16^,^24^,^25^. Like flavin monooxygenases (FMO in plants and animals), YUC proteins contain conserved motifs for binding FAD (flavin-adenine dinucleotide) and NADPH (the reduced form of nicotinamide adenine dinucleotide phosphate)^10^. So far such enzyme activities have been confirmed for 3 YUC proteins (YUC2/4/6)^13^,^14^. Detailed catalytic mechanism have been shown for YUC6^12^. YUC6 uses NADPH to catalyze the reduction of FAD to FADH^−^; FADH^−^ then reacts with oxygen to form a flavin-C4α- (hydro) peroxy intermediate, which reacts with IPyA to produce IAA^12^.

To provide evidence that CKRC2/YUC8 is also capable of catalyzing the conversion of IPyA to IAA, the *CKRC2/YUC8* cDNA was cloned into the expression vector pET28b^+^ and the protein was expressed in a cell-free protein expression system. The enzymatic activities of the expressed YUC proteins were analyzed (Fig. 3d & Supplementary Fig. S7)^27^. The results showed that YUC1 and YUC8 proteins could convert 54.28% and 50.34% of total IPyA into IAA, respectively (Fig. 3d) whereas in the control cells expressing Renilla luciferase only a small amount of IPyA was converted to IAA non-enzymatically (Fig. 3d). Hence as reported for other YUC proteins^12^,^13^,^14^ YUC8 is capable of catalyzing the conversion of IPyA to IAA.

### CKRC1/TAA1 and CKRC2/YUC8 are involved in the regulation of auxin biosynthesis by CK

**Figure 4:**
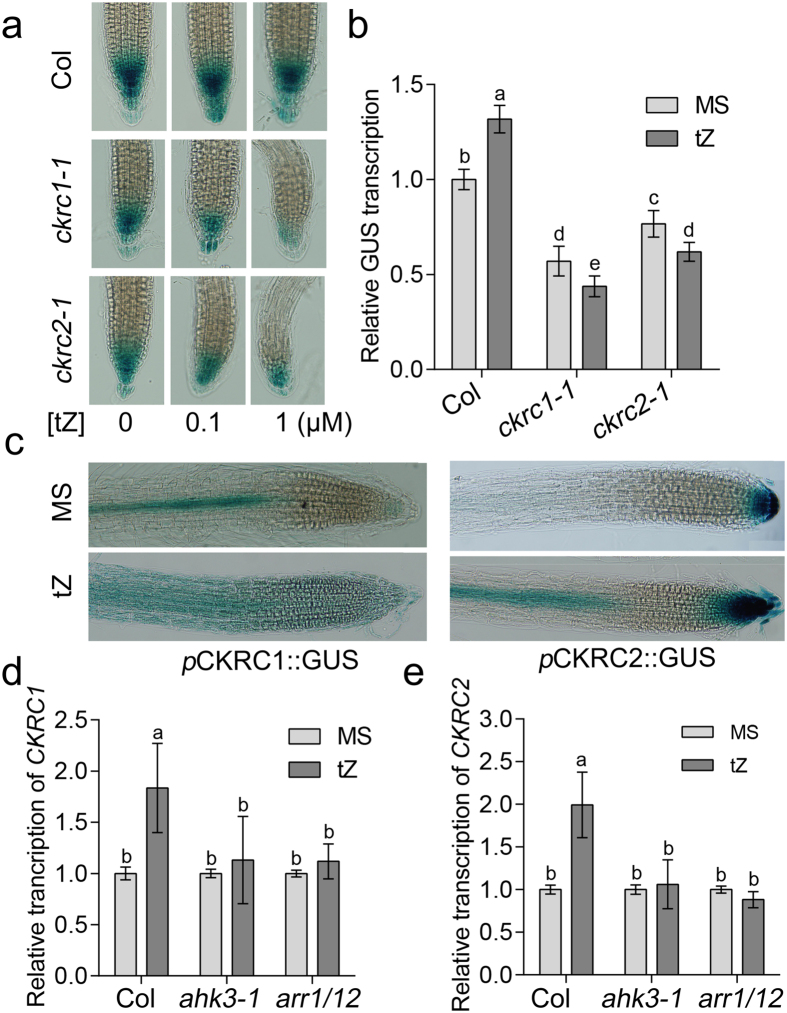
The IPyA pathway catalyzed by CKRC1/TAA1 and CKRC2/YUC8 is involved in CK induced auxin biosynthesis in roots. Dr5::GUS expression (**a**) and transcription (qRT-PCR) (**b**) in root tips of wildtype Col plants as well as *ckrc2-1* and *ckrc1-1* mutants, shows the reduction of GUS activity in the two mutants after tZ treatment; (**c**) The GUS staining of *pCKRC1::GUS* and *pCKRC2::GUS* transgenic roots increases after tZ treatment; (**d**,**e**) The AHK3-ARR1/12 signalling pathway is involved in the process of induction of *CKRC1* (**d**) and *CKRC2* (**e**) by CK. The seedlings were grown on MS medium with or without 0.1 μM tZ for 7d and their roots were used for RNA extraction. Each staining shown in (**a**,**c**) represents data on at least 20–30 roots. Data show the mean of 3 biological replicates. Error bars indicate ± SD. Different letters indicate significant differences at *P* < 0.05 according to ANOVA followed by Tukey’s multiple comparison tests.

The interaction between auxin and CK plays a key role in plant growth and development. Recently, it was suggested that the IPyA pathway catalyzed by TAA/TAR and YUC enzymes was the main pathway for auxin biosynthesis in *Arabidopsis*^14^,^16^,^24^. Our previous studies reveal that the ARABIDOPSIS HISTIDINE KINASE3 (*AHK3*)/ARABIDOPSIS RESPONSE REGULATOR1 (*ARR1*)/*ARR12* involved in CK signaling can stimulate auxin biosynthesis via up-regulating the transcription of *CKRC1/TAA1* and other auxin biosynthetic genes including *YUC8*^21^. However, Dr5::GUS activity after CK treatment was reported to be decreased in the root tips of *ckrc1* mutants but not changed significantly in WT root tips, a phenomenon suggested to be due to the dual effect of CK on auxin biosynthesis (positive) and polar transport (negative)^21^. Interestingly, a similar decrease in activity was also observed in *ckrc2-1* mutant after tZ treatment (Fig. 4a,b) suggesting a similar role for *CKRC2/YUC8* in auxin-CK crosstalk. Promoter-GUS staining and qRT-PCR analysis revealed that, like for *CKRC1/TAA1* (Fig. 4c,d), the up-regulation of *CKRC2/YUC8* was impaired in *ahk3-1* and *arr1-3/12-1* mutants (Fig. 4d,e). Therefore, CK can stimulate auxin biosynthesis in roots by up-regulating both *CKRC1/TAA1* and *CKRC2/YUC8* genes via the AHK3-ARR1/12 signaling pathway.

### PIF4 is required for the regulation of auxin biosynthesis by CK

**Figure 5:**
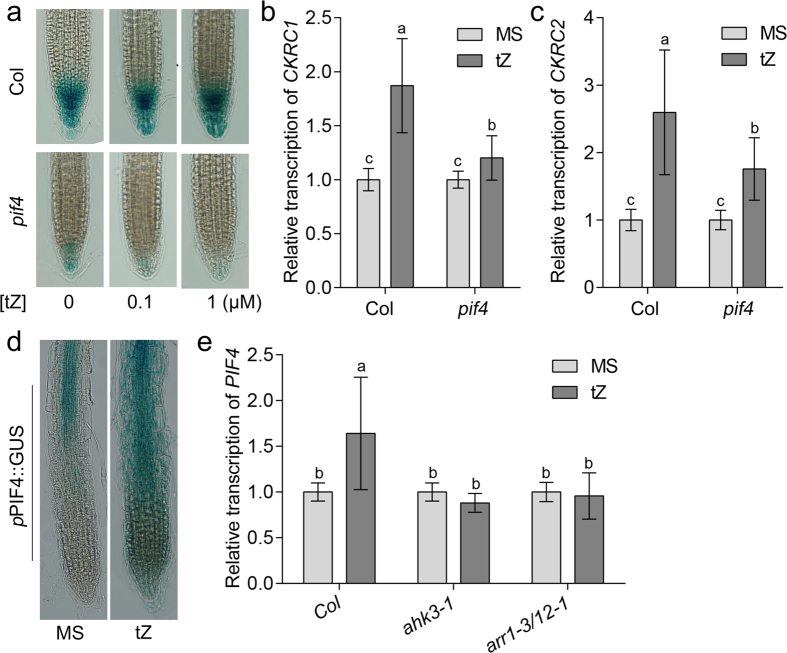
PIF4 is required for the regulation of auxin biosynthesis by CK. (**a**) Dr5::GUS expressions in Col and *pif4* after tZ treatment; Effects of CK on the transcriptions of *CKRC1* (**b**) and *CKRC2* (**c**) genes in WT and *pif4*; (**d**) GUS staining of *pPIF4::GUS* on MS medium with or without tZ; and (**e**) Relative transcription of *PIF4* in Col and different CK signaling mutants. The roots of 7-day-old seedlings grown on MS medium with or without 0.1 μM tZ were used for qRT-PCR. Each staining shown in (**a**,**d**) are representative images of at least 20–30 roots. Data are mean values of 3 replicates. Error bars indicate ± SD. Different letters indicate significant differences at *P* < 0.05 according to ANOVA followed by Tukey’s multiple comparison tests.

Auxin biosynthesis can be regulated by many factors, such as CK^20^,^21^,^28^,^29^, ethylene^30^,^31^, jasmonate^32^,^33^,^34^, temperature^35^,^36^,^37^ and light^38^,^39^. Both CK and high temperature can stimulate auxin biosynthesis by up-regulating the transcription of *CKRC1* and *CKRC2* in roots (Fig. 4c–e & Supplementary Fig. S9), and the effects of temperature are reported to be mediated by phytochrome-interacting factor 4 (PIF4). PIF4 acts as a transcription factor to promote the transcription of *CKRC1* and *CKRC2*^35^,^36^,^37^. To explore whether PIF4 also plays a role in the CK-dependent regulation of auxin biosynthesis, the CK-mediated inductions of *CKRC1* and *CKRC2* in the loss-of-function mutant of *PIF4* (*pif4*) were compared with the wild-type Col-0. The results showed that *CKRC1* and *CKRC2* genes were no longer induced obviously in *pif4* (Fig. 5b,c) indicating that PIF4 is essential for transcriptional regulation of *CKRC1* and *CKRC2* by CK. As for *ckrc1* and *ckrc2* the relative *Dr5::GUS* expression in *pif4* mutants after tZ treatment was significantly reduced (Fig. 5a). Importantly, we found that tZ treatment also had a positive effect on the transcription of *PIF4* suggesting that *PIF4* itself is regulated by CK (Fig. 5d,e). qRT-PCR analysis showed that the induction of *PIF4* by tZ is impaired in the CK-signaling mutants *ahk3* and *arr1*/*12* (Fig. 5e). Taken together, these results put the transcriptional factor PIF4 between AHK3-ARR1/ARR12 signaling and the transcriptional induction of *CKRC1*/*2* for mediating CK-dependent regulation of auxin biosynthesis. However, the effect of the *pif4* mutant was much weaker compared to the responses of *ckrc1-1* and *ckrc2-1* mutants to tZ and IAA treatment (Supplementary Figs S10 and S11). This might be due to the fact that in *pif4* mutant roots the CKRC1-CKRC2 catalyzed IPyA pathway is functional and thus this mutant has more locally synthesized IAA than the *ckrc1-1* or *ckrc2-1* mutants. A comparison of the CK-induced *CKRC1/2* transcription between WT and the mutants *pif5, pif7* and *erf109* -these proteins are reported to have binding activity to the *YUC8* promoter to mediate responses to temperature^35^,^36^,^37^ or jasmonates^32^ - revealed no significant roles of these three proteins in CK regulation (Supplementary Fig. S12).

**Figure 6:**
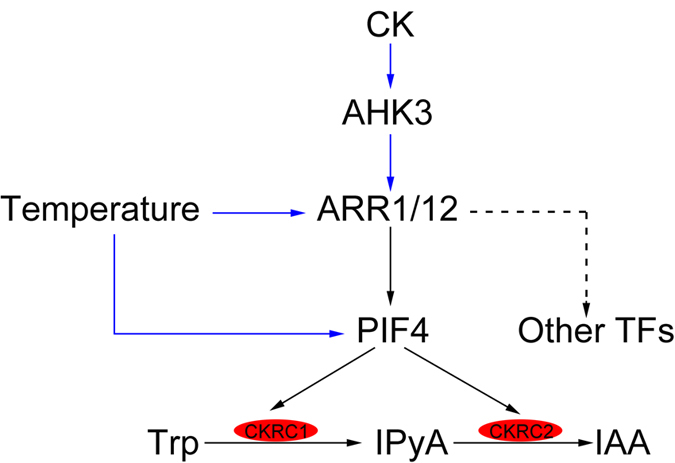
Diagram of temperature and CK regulated auxin biosynthesis in roots. Black lines indicate the results obtained from this article; blue lines indicate the results obtained from previous studies by other researchers; and dashed line stands for the deduction.

In summary, *CKRC2* encodes a previously identified member of YUCCA (YUC) flavin monooxygenase-like proteins (YUC8) and is expressed abundantly in roots. Like other YUCs, CKRC2/YUC8 is a rate-limiting enzyme for catalyzing the conversion of IPyA to IAA and is acting downstream of CKRC1/TAA1 in the IPyA pathway. The transcription of both *CKRC1/TAA* and *CKRC2*/*YUC8* genes can be induced by CK, and the phytochrome-interacting factor 4 (PIF4) is required for this regulation via the classical AHKs-ARR1/12 signalling pathway. Thus, PIF4 plays an essential role in mediating the regulatory effect of CK on the transcription of *CKRC1* and *CKRC2* genes in the IPyA pathway of auxin biosynthesis. The appearance of an ARR1 recognition sequence (5′-AGATT-3′) in the 600 bp region upstream from the *PIF4* translation start site implies that *PIF4* may be directly targeted by ARR1^40^. This possibility should be tested by yeast one-hybrid or Chip analysis in the future. These results together with previously reported data^30^,^31^,^35^,^36^,^37^,^38^,^39^, suggest that PIF4 is a central factor to mediate the regulation of auxin biosynthesis (Fig. 6).

## Methods

### Plant material and growth conditions

For mutant screening, the *Arabidopsis thaliana* activation-tagged T-DNA pools (CS31100, Col-2 background, composed of approximately 62,000 individual lines)^41^, were purchased from the Arabidopsis Biological Resource Center (ABRC) ( http://abrc.osu.edu/). Germination and plant growth was carried out at 25 °C with a 1 h light/8 h dark cycle. For growth analyses, seedlings were grown for 7 days on vertical Miller-Skoog plates (MS) (1.1% w/v agar and 10 g/L sucrose)^21^,^42^.

*Arabidopsis* accession Col-2 was used as wild-type control. The following mutants have been used in this study: *pDR5::GUS* marker line^43^; *ckrc1-1* (*ckrc1-1/pDR5::GUS*; *At1g70560*)^21^,^22^, *ckrc2-2* (N655757; *At4g28720*), *yucca1* (N655809; *At4g32540*), *yucca2* (N659779; *At4g13260*), *yucca4* (N104041; *At5g11320*), *yucca6* (N663363; *At5g43890*), *yucca7* (N659416; *At2g33230*), *yucca11* (N573485; *At1g21430*), *arr1-3/12-1* (N6981; *At3g16857/At2g25180*) and *ahk3-1* (*N6562; At1g27320*) were purchased from the The Nottingham Arabidopsis Stock Centre (NASC); Seeds for *yucca3* (GABI_376G12; *At1g04610*), *yucca5* (CSHL_GT6160; *At5g43890*), *yucca9* (SAIL_762-D07; *At1g04180*) and *yucca10* (FLAG_599G05; *At1g48910*) were kindly provided by Prof. Yunde Zhao (University of California, San Diego); *p*YUC8::GUS (line 132.1/132.2) and *p*35S::YUC8 (line 027.10.1W) by Prof. Stephan Pollmann (Campus de Montegancedo, Madrid) and the double homozygous *pif4/pDR5*::^GUS^ (AGI codes: *At2g43010*) mutants by Prof. Chuanyou Li (Institute of Genetics and Developmental Biology, Chinese Academy of Sciences)^37^.

To generate *ckrc2-1/p*DR5::GUS mutants, the *ckrc2-1* mutant was crossed with Col/DR5::GUS and the double homozygous mutant in F2 was identified by the non-segregation of GUS staining in its seeds (F3).

### Plant Vectors and Transformations

For constructing *p*CKRC2::*CKRC2*, the *CKRC2* cDNA with stop codon and promoter were introduced into pCAMBIA1300 binary vector. The primers used for cloning *pCKRC2*::*CKRC2* were YUC8-F/R (Supplementary Appendix S1). For *pCKRC1*::*CKRC2*, the full length cDNA was amplified using YUC8-cDNA-F/R (Supplementary Appendix S1) and introduced into p*CKRC1*::*GUS* vector^21^. After sequencing confirmation ( http://www.genomics.cn/), the constructed binary vectors were introduced into either wild-type Col plants (for *pCKRC1*::*CKRC2*) or *ckrc2-1* mutant plants (for *pCKRC*1::*CKRC2* and *pCKRC2*::*CKRC2*) via Agrobacterium tumefaciens-mediated (strain GV3101) floral-dip transformation method^44^. Selection for transgenic plants was performed as described in previous studies^21^.

For CKRC2 protein expression vectors, the *CKRC2* cDNA without stop codon were introduced into the vector pET28b^+^. The primers used for amplifying the YUC8 cDNA were YUC8-P-R/F (Supplementary Appendix S1). After confirming the amplified cDNA by sequencing, vectors were transformed into *E. coli* DH5α for analysis of the enzymatic activity.

### Phenotype characterization

For root inhibition assays and biochemical complementation, seeds were germinated and grown vertically on MS medium with various hormones or compounds at 25 °C with a 16/8 h light/dark cycle for 7 days. Data shown are the mean values of three separate experiments using at least 40 seedlings.

For analyzing root gravitropism germinated seedlings were transferred to fresh MS media and grown vertically on MS plates 5 days at 25 °C with a 16/8 h light/dark cycle. Three hours later, the plates were rotated 90 degrees, further incubated for 24 h and the degree of gravitropic response was measured for each root. Approximately 100 seedlings were measured for each genotype and treatment.

### TAIL-PCR, genetic mapping and identification of mutated gene

TAIL-PCR was performed according to Liu *et al.*^45^. In brief, tail-PCR was used to clone the T-DNA flanking sequences in isolated mutants^45^,^46^. All PCR products were electrophoretically separated on a 1% agarose gel and the expected TAIL-3 products were purified and sequenced. DNA sequences were aligned with Blastn ( http://www.ncbi.nlm.nih.gov/BLAST/) and Tair10 ( http://www.arabidopsis.org/Blast/) software. Map-based cloning was performed using F2 populations generated by backcrossing the *ckrc2-1* mutant (Col background) with Landsberg (Ler) ecotype wild-type plants, as previously described^47^. High-throughput sequencing (ShangHai Biotechnology Corporation, China, http://www.shbiotech.org/ and Hangzhou Guhe Information and Technology Co., Ltd, China, http://www. guheinfo.com/) was used to reveal the exact DNA mutation in the mapped region of the mutation.

### RNA preparation and real-time qRT-PCR analysis

RNA was extracted using Trizol agent (Sangon, http://www.sangon.com/). Reverse transcription was performed by a reverse transcription kit (DRR047A) (Takara, http://www.takara-bio.com/). For synthesizing first-strand cDNA 1 μg of total RNA was used after the pretreatment by RNase free-DNase. The cDNA was diluted 5 times for Real-Time PCR.

For quantitative RT-PCR (qRT-PCR) 20 μL amplification reactions (10 μL SYBR Premix Ex Taq (Takara, http://www.takara-bio.com/), 0.8 μL of each primer (Supplementary Appendix S1), 1.6 μL cDNA and 6.4 μL ddH_2_O) were used. The results of each primer pair were normalized relative to ACTIN 8 (AGI codes: *At1g49240*). All realtime qRT-PCR amplifications were performed in a Bio-Rad CFX96^TM^ Real-time System (Bio-Rad, http://www.bio-rad.com). The following PCR program was used: An initial denaturation at 95 °C for 30 s; 40 cycles of 95 °C for 5 s and 60 °C for 30 s. During the melting curve analysis, PCR reactions were denatured at 95 °C for 15 min. Each experiment was repeated three times and each reaction was performed in triplicates^48^.

For analyzing the CK- and auxin-induced *ARR5/15* (AGI codes: *At3g48100/At1g74890*) and *IAA1/2* (AGI codes: *At4g14560/At3g23030*) transcription, 7-day-old seedlings grown on MS medium were treated in liquid MS medium with 10 μM trans-zeatin (tZ) for 30 minutes or 20 μM IAA for 1.5 hours^49^,^50^.

For analyzing the CK induced gene transcription, 7-day-old seedlings grown on MS and/or on 0.1 μM tZ medium were used.

### Histochemical GUS assay

7-day-old seedlings containing a *GUS* marker were grown on medium with or without 0.1 μM tZ and then incubated in 1 mM X-gluc (5-bromo-4-chloro-3-indolyl-β-D-glucuronide) and 50 mmol/L potassium phosphate buffer, pH7.5, with 0.1% v/v Triton X-100 for GUS staining at 37 °C for 45 minutes (Col/Dr5::GUS, *ckrc1-1*/Dr5::GUS, *ckrc2-1*/Dr5::GUS and *pif4*/Dr5::GUS), 1 h (*p*CKRC1::GUS) and 3 h (*p*CKRC2::GUS and *p*PIF4::GUS).

### Cell-free protein expression and enzyme activity tests

For protein expression, a S30 T7 High-Yield Protein Expression System (Promega) was used according to the manufacturer’s instructions. This kit contains all components used for expressing proteins in prokaryotic expression system and plasmid DNA encoding Renilla Luciferase as control protein.

To test the enzyme activities about 2 μg protein (YUCCA1, CKRC2 and Renilla Luciferase) (the amount was estimated from SDS-PAGE by comparison with the protein marker), NADPH (50 mM) 20 μL, FAD (2 mM) 2 μL, IPyA (50 mM) 0.4 μL and add nuclease-free water to a final volume of 100 μL. The mixture was incubated at 30 °C for 2 hours with vigorous shaking. For quantitative analysis, the reaction mixture was diluted 100 times before the measurement. The fluorescence intensities were measured at λex/λem = 363 nm/277 nm in a 1 cm quartz cell and with a slit at 2 nm for the excitation and 5 nm for the emission. Scan speed were 350 nm/min. The fluorescence values were quantified using a standard curve (y = 380.8x + 36.31).

## Additional Information

**How to cite this article**: Di, D.-W. *et al.* Functional roles of Arabidopsis *CKRC2/YUCCA8* gene and the involvement of PIF4 in the regulation of auxin biosynthesis by cytokinin. *Sci. Rep.* **6**, 36866; doi: 10.1038/srep36866 (2016).

**Publisher’s note:** Springer Nature remains neutral with regard to jurisdictional claims in published maps and institutional affiliations.

## Supplementary Material

Supplementary Information
