# Supplementary material for: Functional roles of Arabidopsis CKRC2/YUCCA8 gene and the involvement of PIF4 in the regulation of auxin biosynthesis by cytokinin
Source: Sci Rep. 2016 Nov 9;6:36866. doi: 10.1038/srep36866 (PMC5101810; doi:10.1038/srep36866)
Supplement: Supplementary Information [file srep36866-s1.pdf]

Functional roles of Arabidopsis *CKRC2/YUCCA8* gene and the involvement of PIF4 in the regulation of auxin biosynthesis by cytokinin

Dong-Wei Di[1, §], Lei Wu[1], Li Zhang[1], Chen-Wei An[1], Tian-Zi Zhang[1], Pan Luo[1], Huan-Huan Gao[1], Verena Kriechbaumer[2] and Guang-Qin Guo[1,*]

[1.] Institute of Cell Biology and MOE Key Laboratory of Cell Activities and Stress Adaptations, Lanzhou University, Lanzhou 730000, China

[2.] Plant Cell Biology, Biological and Medical Sciences, Oxford Brookes University, Oxford OX3 0BP, United Kingdom

[§] Present address: Institute of Soil Science, Chinese Academy of Sciences, Nanjing 210008, China

[*] Author for correspondence (gqguo@lzu.edu.cn)

Supplemental Data

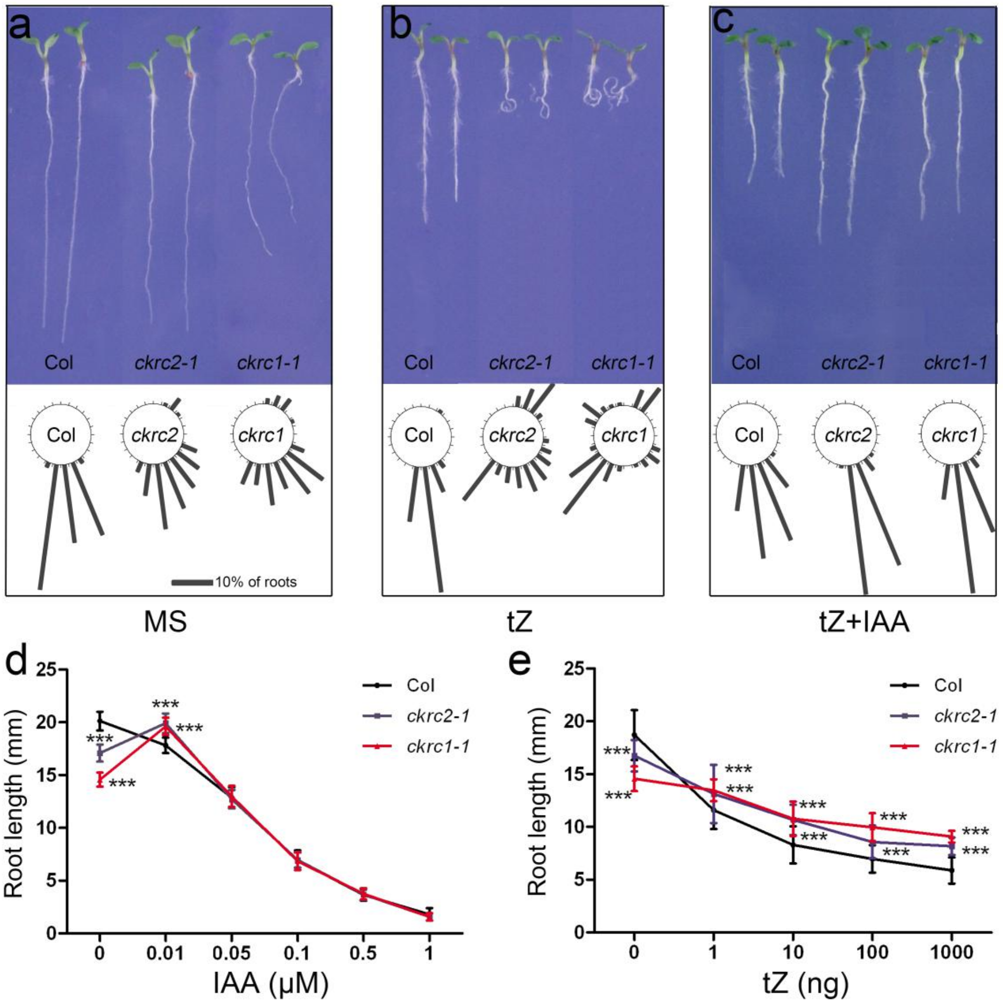

Figure S1. ***ckrc2-1*** **exhibits altered response to CK and auxin.**

(a-c) *ckrc2-1* roots exhibited defective gravitropic response on MS (a), which was more serious on the medium containing 0.1μM tZ (b) and can be rescued by 0.01μM IAA (c). (n=75-100). (d and e) Col and *ckrc2-1* mutant were grown on the medium with various concentrations of IAA (d) and tZ (e) and the primary root elongation was measured after germination 7d. All experiments were repeated 3 times. Shown are means ± SD with n=40-60 in each repeat. $^{***}P < 0.001$.

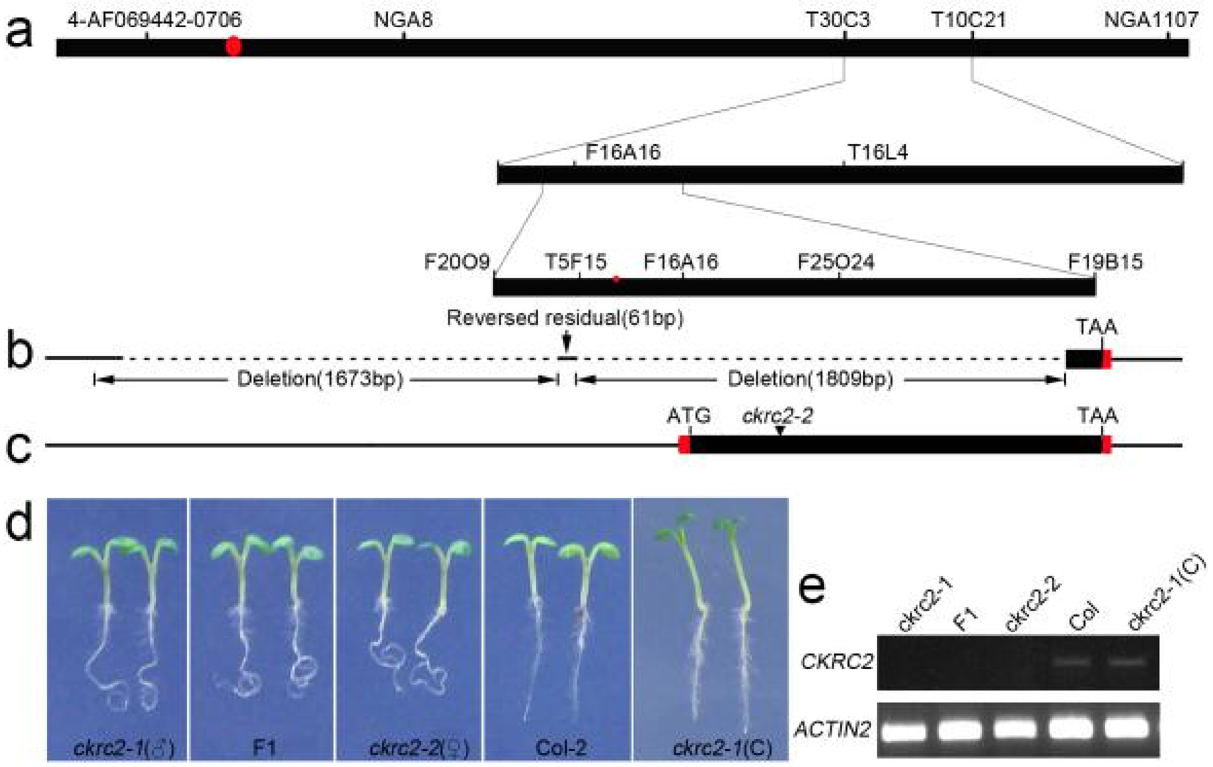

Figure S2. Map-based identification of *CKRC2* gene.

(a) Genetic mapping; (b-c) The deletions in *ckrc2-1* (b) and the mutant alleles of *CKRC2* (c); (d) *ckrc2-1* is an allele mutant of *YUC8*; (e) RT-PCR analysis on the transcription levels of *CKRC2*/*YUC8* in *ckrc2* mutants, WT and the mutant complemented by *YUC8* [*ckrc2-1*(C)].

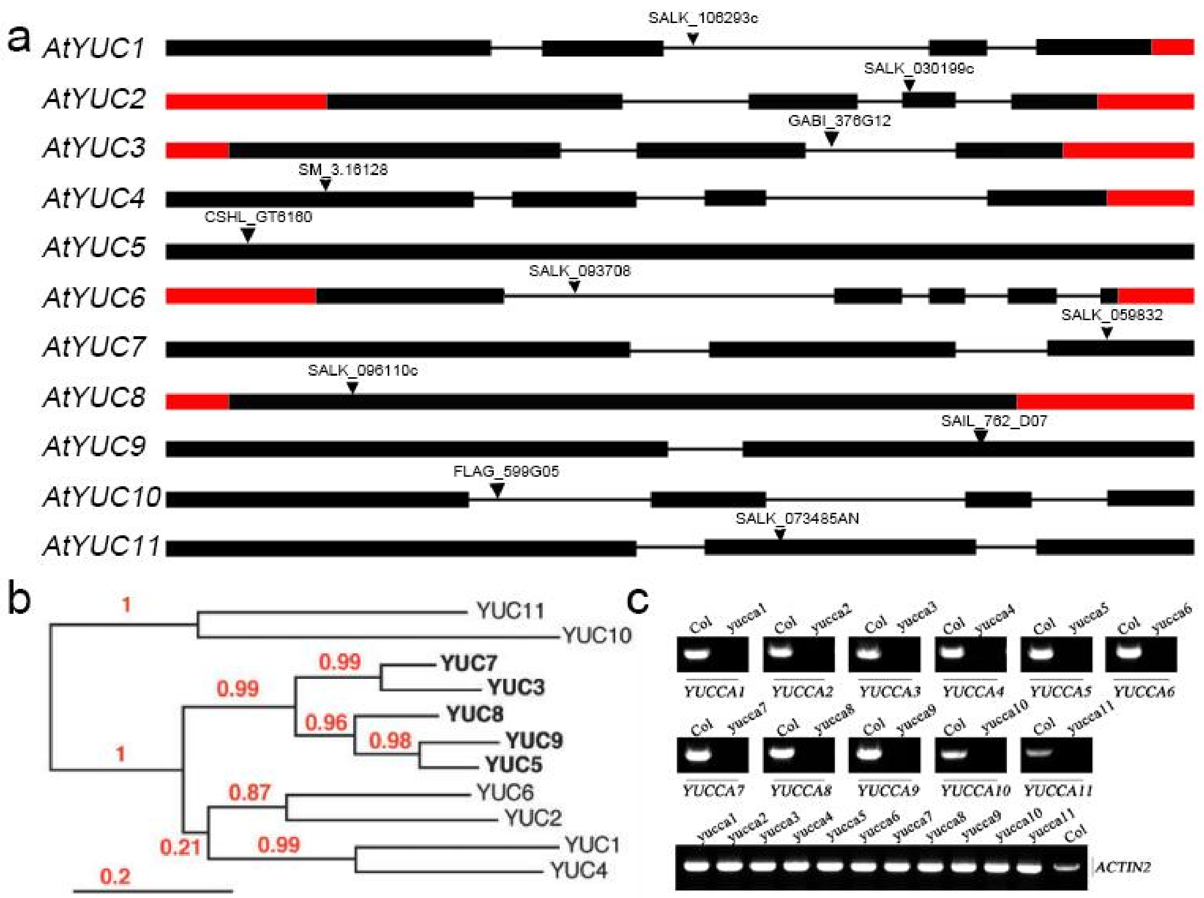

Figure S3. The gene structures (a) **and phylogenetic relationships** (b) **of YUC family**

(Cheng et al., 2006; Chen et al., 2014); Locations of T-DNA insertions in each *yuc* mutants are indicated and their homozygous state are confirmed by RT-PCR (c).

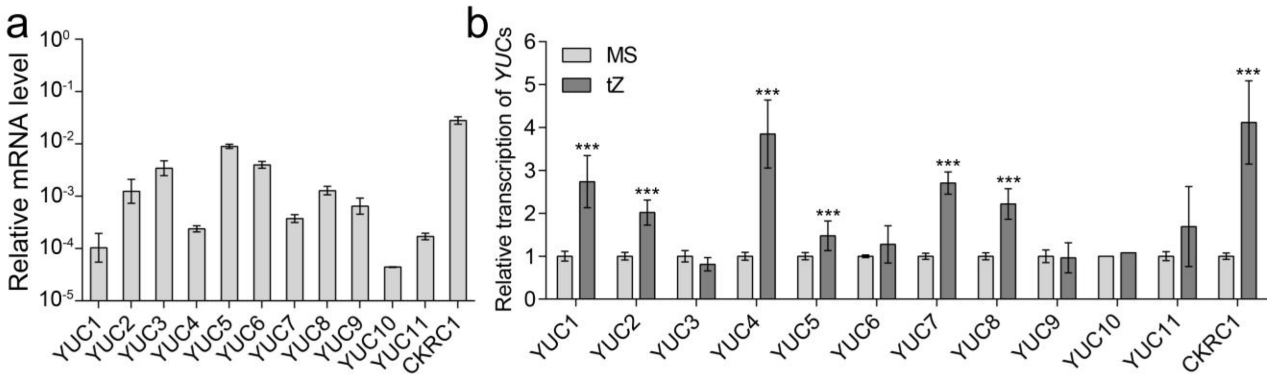

Figure S4. The relative transcription (a) **of** *YUC* genes **and their induction level by 0.1 μM tZ** (b) **in whole seedlings.**

Data are mean of 3 replicates. Error bars indicate SD. ***P<0.001.

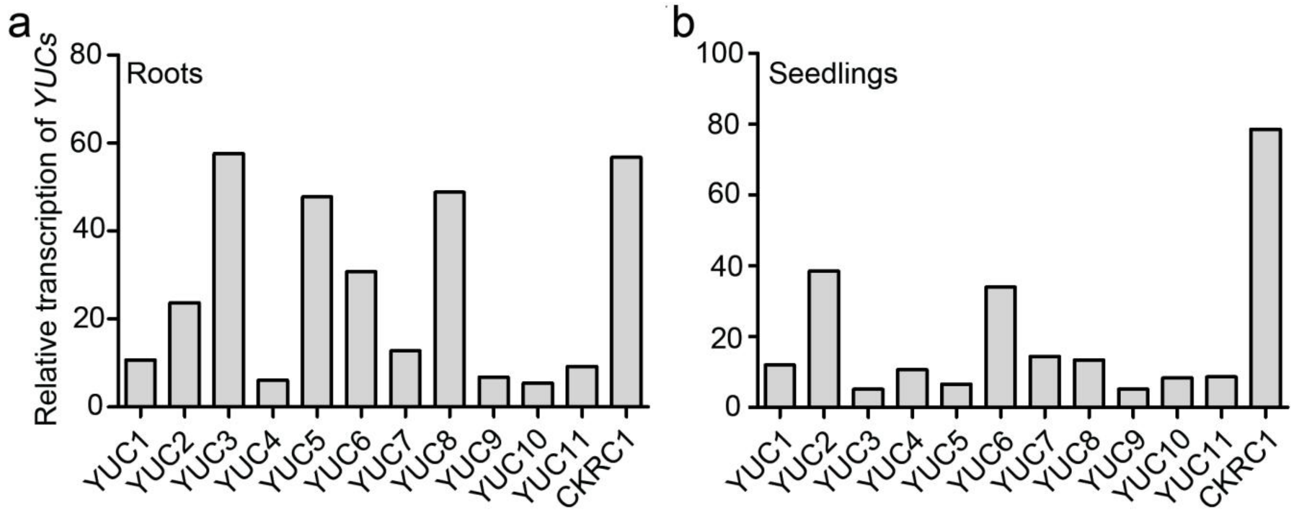

Figure S5. The relative transcription of *YUC* in root (a) **and seedlings** (b)**.**

Data retrieved from the AtGenExpress Visualization Tool (http://jsp.weigelworld.org/expviz/expviz.jsp).

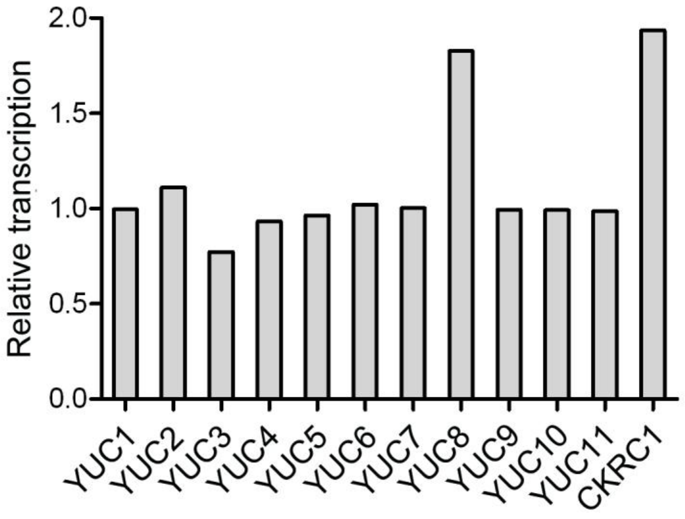

Figure S6. Effect of tZ treatments on the transcription of *YUC* family and *CKRC1/TAA1*.

The seedlings were treated by 1 μM tZ 0.5 hours before RNA extraction. Data retrieved from the AtGenExpress Visualization Tool (http://jsp.weigelworld.org/expviz/expviz.jsp).

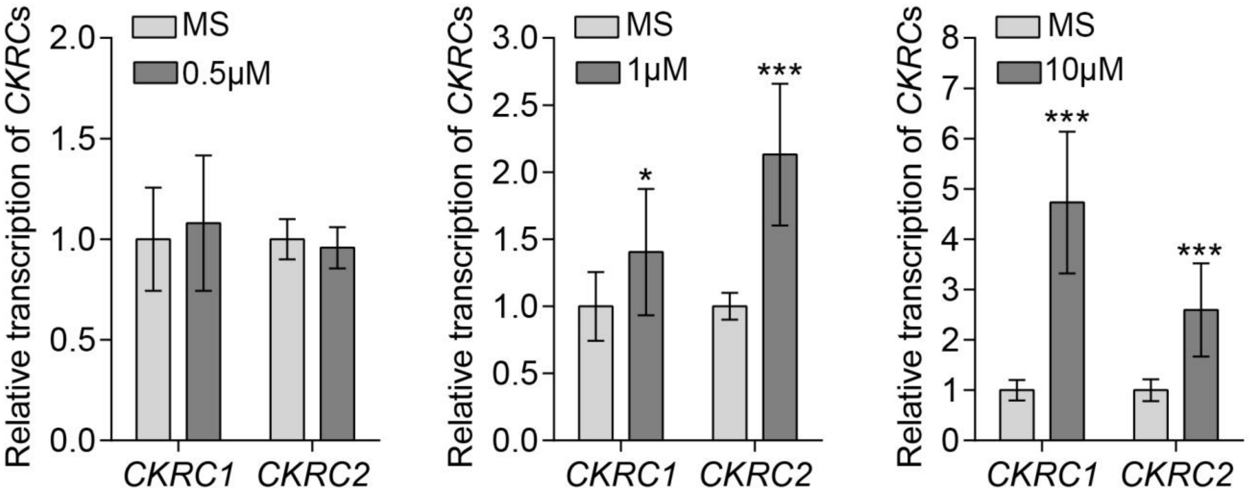

Figure S7 The relative transcription of *CKRC1* and *CKRC2* after short-term tZ treatment in whole seedlings.

Data are mean of 3 replicates. Error bars indicate SD. *P < 0.05 ; ***P < 0.001.

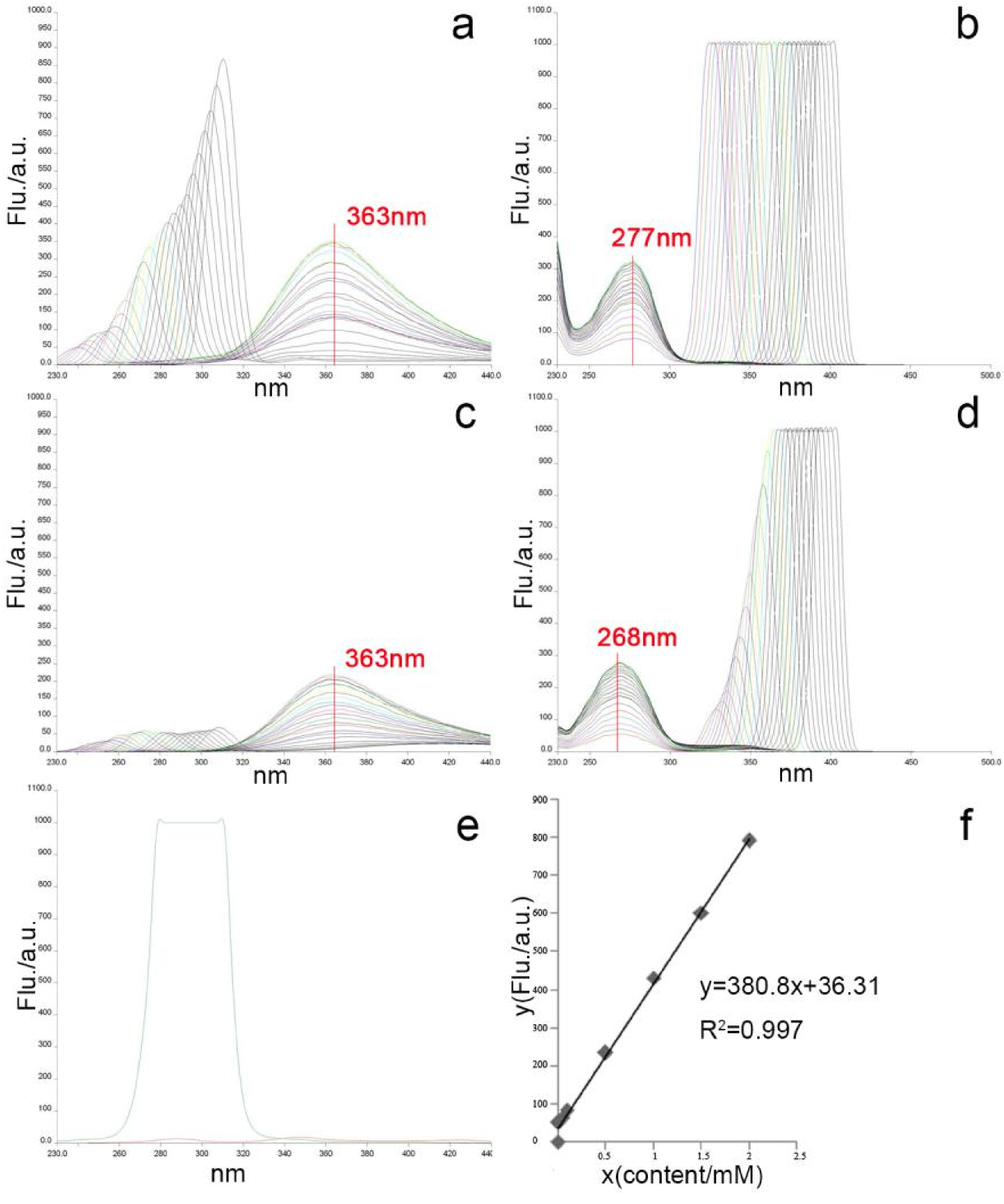

Figure S8. Emission and excitation spectra of IAA and IPyA.

Identification the emission spectrum of IAA (a) and IPyA (c), and excitation spectrum of IAA (b) and IPyA (d) by 3-D fluorescence analysis; (e) Distinguishing the IAA and IPyA by constant wavelength synchronous luminescence (△λ=35nm); (f) Standard curve of IAA.

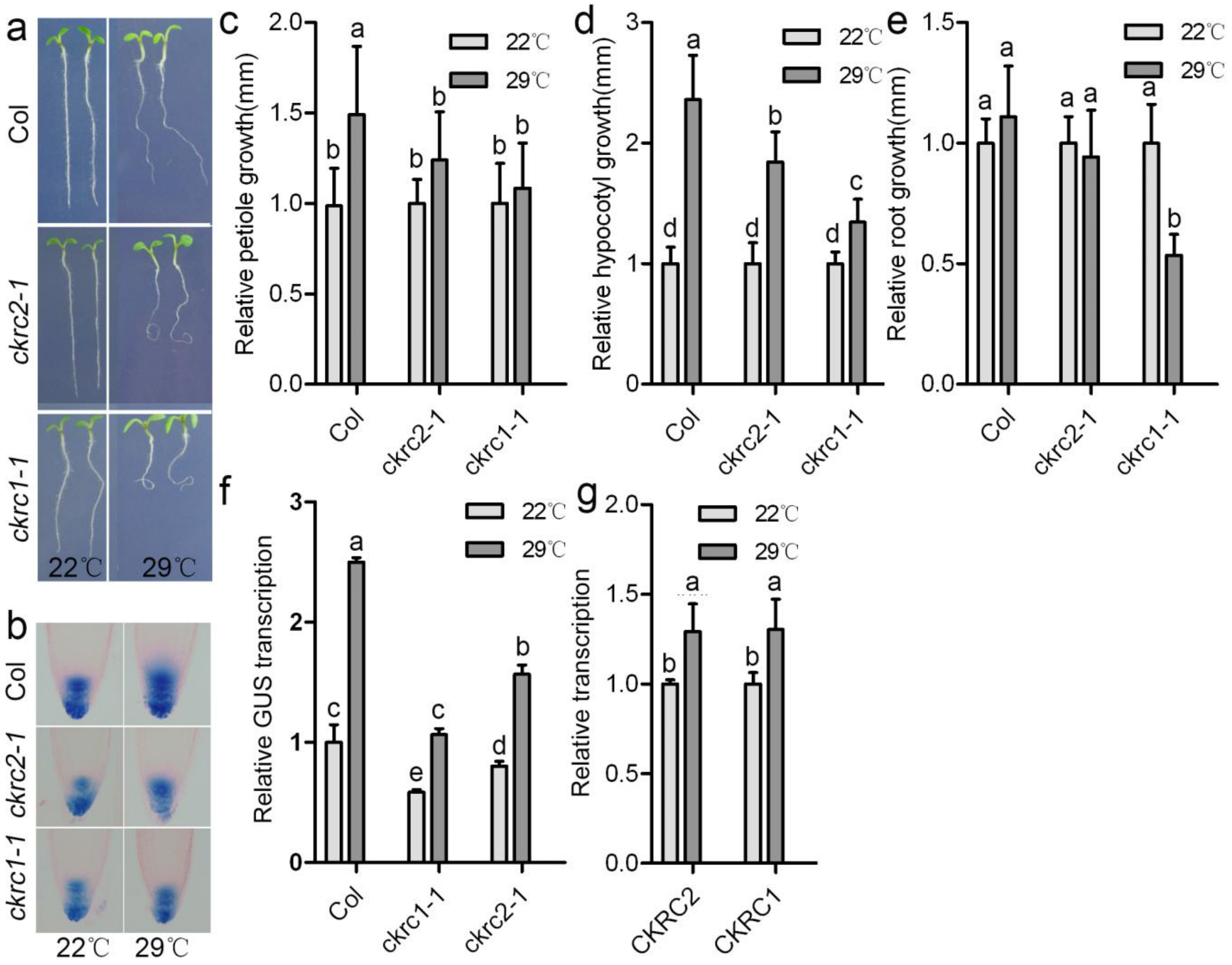

Figure S9. The auxin-related responses to temperature are dependent on CKRC1-CKRC2 pathway.

(a) Root phenotype; (b) GUS-staining (c-e) The relative length of petioles (c), hypocotyls (d), and roots (e). Each experiment was repeated three times. Shown are means ± SD with n=40-45 in each repeat; (f-g) Effects of temperature on the transcription of *GUS* gene (f) and *CKRC1/CKRC2* (g). Data are mean of 3 replicates. Error bars indicate ± SD. Different letters indicate significant differences at $P < 0.05$ according to ANOVA followed by Tukey's multiple comparison tests.

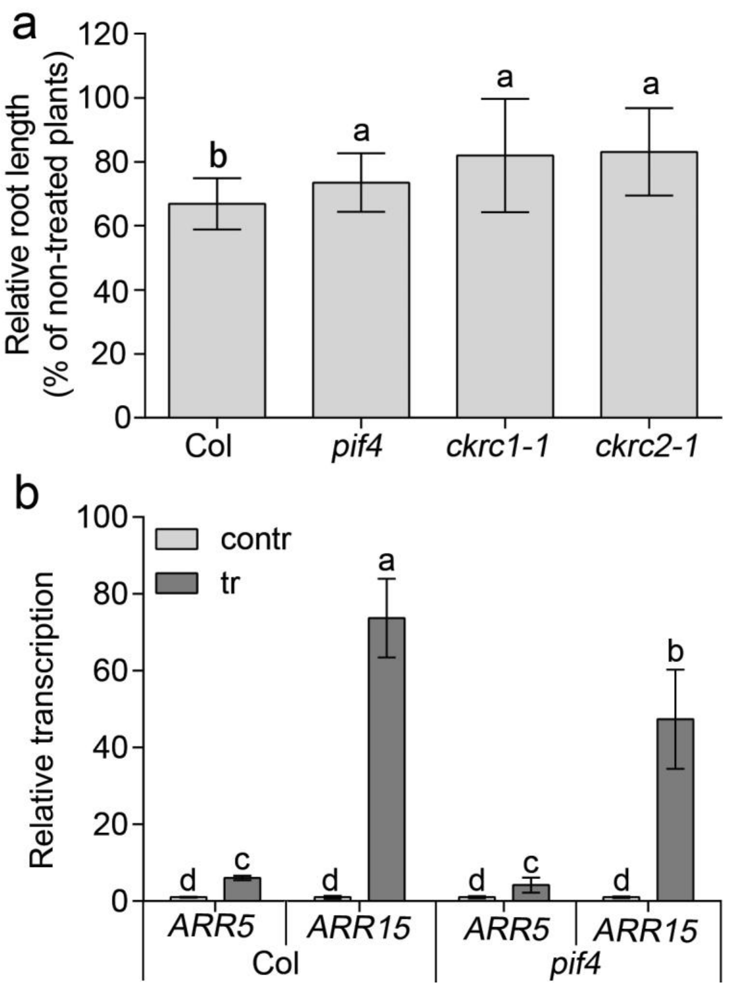

Figure S10. The response to CK in *pif4* mutant.

(a) Col and *pif4* mutant were grown on the medium with 0.1 μM tZ and the primary root elongation was measured after germination 7d. All experiments were repeated 3 times. Shown are means ± SD with n=40-60 in each repeat. (b) *pif4* mutant has the normal response to exogenous CK. Data are mean of three biological replicates (n =3). Error bars indicate ± SD. Different letters indicate significant differences at P < 0.05 according to ANOVA followed by Tukey's multiple comparison tests.

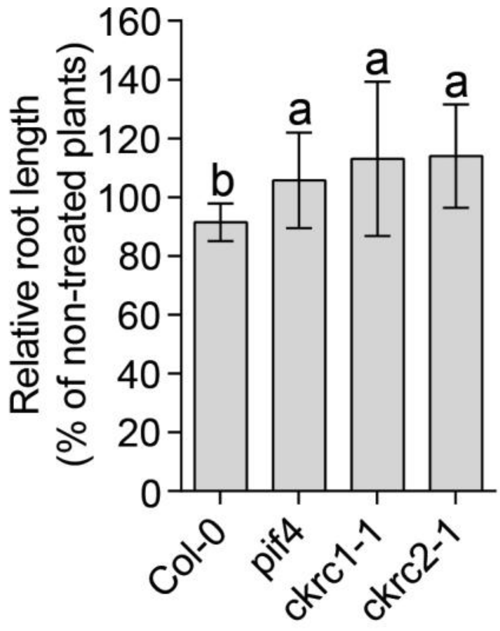

Figure S11. The root length of *pif4* and Col grown on medium with various concentrations of IAA.

Col and *ckrc2-1* mutant were grown on the medium with 0.01 μM IAA and the primary root elongation was measured after germination 7d. All experiments were repeated 3 times. Shown are means ± SD with n=40-60 in each repeat. Different letters indicate significant differences at $P < 0.05$ according to ANOVA followed by Tukey's multiple comparison tests.

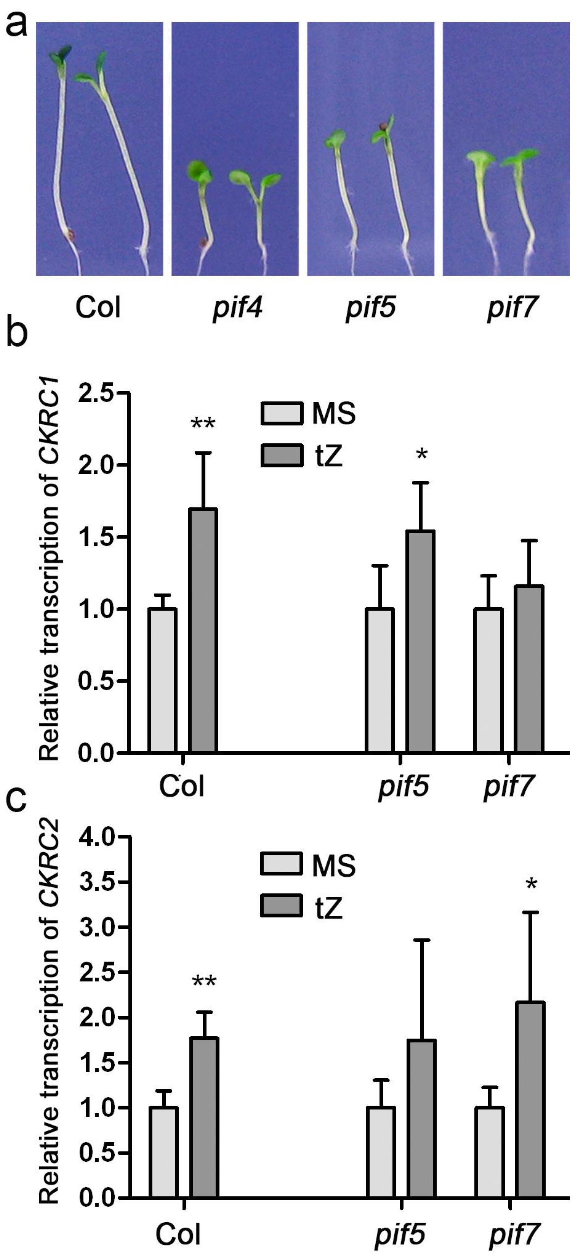

Figure S12. PIF5 and PIF7 may not influence *CKRC1* and *CKRC2* transcription after tZ treatment.

(a) Phenotypes of 4-days-old Rc-grown WT and *pif*s mutants. Rc influence rate was μmol·m^{-2}·s^{-1}. Effects of CK on the transcriptions of *CKRC1* (b) and *CKRC2* (c) genes in WT and *pif*s mutants. The roots of 7-day-old seedlings grown on MS and 0.1 μM tZ medium were used for qRT-PCR. Data were means of 3 replicates. Error bars indicated ± SD. 0.01 < *P < 0.05; **P < 0.01.

Appendix S1 PCR primer sequences

For Real-Time PCR:

QRT-ACT8 F	TGTGACAATGGTACTGGAATGG
QRT-ACT8 R	TTGGATTGTGCTTCATCACC
QRT-CKRC1 F	AGATGATGAAGAATCGGTGGG
QRT-CKRC1 R	ACGCAAACGCAGGGTAAGA
QRT-YUC1-F	CGTGGAGTCCCTTCTTTGAT
QRT-YUC1-R	TCTGAGTCGGTCGTAGGTTT
QRT-YUC2-F	ATTGTCTCATCCTTCTTCCCTC
QRT-YUC2-R	TTCGTCCAATACCTTGAGTCTTAC
QRT-YUC3-F	AACGCTGAGAAAGTTGTGCC
QRT-YUC3-R	GATGGGTTTGCTCCGTGATT
QRT-YUC4-F	TTTCACGGCTTGCGTCACTT
QRT-YUC4-R	CGTTGGGCAATACCGACCTT
QRT-YUC5-F	CATTAGCATTGTGATTTGCGAGAT
QRT-YUC5-R	GGAGTTTGAAGGCGAGGTGA
QRT-YUC6-F	CAAACGCAGCCGACTCAACC
QRT-YUC6-R	CCTACCTACCCGACGAAGCA
QRT-YUC7-F	CCAGCCTTCCCTTTCCACCC
QRT-YUC7-R	TGCTAGACATCGGAGCCATT
QRT-YUC8-F	ACCGCAGCAACGTCCCATAT
QRT-YUC8-R	CTCGTAAACCCAACCGCATA
QRT-YUC9-F	AAGGAGTCCCATTCGTTGTG
QRT-YUC9-R	CGTTGGGTATTCAGGGTAGTG
QRT-YUC10-F	GGAGACCGTAGTGGTGATTG
QRT-YUC10-R	TTTGGCTAAGTGAAGTTTGAGA
QRT-YUC11-F	CAAAGCAATTCTGTCAACTCCCTC
QRT-YUC11-R	CCGTCGCAGCAACCATAAAC
QRT-ARR5F	CCAGTCATCCCAGGCATAGAGT
QRT-ARR5R	GATCGGAAGTTCATCGAGCGG
QRT-ARR15F	GGAGTGTCGTCATCAAGGGAG
QRT-ARR15R	GAGGTGGTGAAGCTGAAGAAGG
QRT-GUS-F	CCCTTACGCTGAAGAGATGC
QRT-GUS-R	GAGGTTAAAGCCGACAGCAG

For vector construction:

YUC8-cDNA-F	GGCGGATCCATGGAGAATATGTTTCGTTTG
YUC8-cDNA-R	GGCGAGCTCTTAGAACTGTTGAGAGATACACCT
YUC8-F	GGCTGCAGTAAGCTTTTCTAGTAACCCAACCCA
YUC8-R	GCGAGCTCTTAGAACTGTTGAGAGATACACCTT
YUC8-P-R	GGCGGCGGATCCATGGAGAATATGTTTCGTTTG
YUC8-P-F	GGCCGAGCTCTTAGAACTGTTGAGAGATACACCT
